# InGaN as a Substrate for AC Photoelectrochemical Imaging

**DOI:** 10.3390/s19204386

**Published:** 2019-10-11

**Authors:** Bo Zhou, Anirban Das, Menno J. Kappers, Rachel A. Oliver, Colin J. Humphreys, Steffi Krause

**Affiliations:** 1 School of Engineering and Materials Science, Queen Mary University of London, Mile End Road, London E1 4NS, UK; b.zhou@qmul.ac.uk (B.Z.); a.das@qmul.ac.uk (A.D.); c.humphreys@qmul.ac.uk (C.J.H.); 2 Department of Materials Science and Metallurgy, University of Cambridge, 27 Charles Babbage Road, Cambridge CB3 0FS, UK; mjk30@cam.ac.uk (M.J.K.); rao28@cam.ac.uk (R.A.O.)

**Keywords:** photoelectrochemistry, InGaN/GaN epilayer, cell imaging, light-activated electrochemistry, light-addressable potentiometric sensor

## Abstract

AC photoelectrochemical imaging at electrolyte–semiconductor interfaces provides spatially resolved information such as surface potentials, ion concentrations and electrical impedance. In this work, thin films of InGaN/GaN were used successfully for AC photoelectrochemical imaging, and experimentally shown to generate a considerable photocurrent under illumination with a 405 nm modulated diode laser at comparatively high frequencies and low applied DC potentials, making this a promising substrate for bioimaging applications. Linear sweep voltammetry showed negligible dark currents. The imaging capabilities of the sensor substrate were demonstrated with a model system and showed a lateral resolution of 7 microns.

## 1. Introduction

Over the past three decades since first being proposed by Hafeman et al. in 1988 [1], photocurrent imaging with light-addressable potentiometric sensors (LAPS) has received increasing attention for chemical and biological applications such as the detection of ions [2], redox potentials [3], enzymatic reactions [4] and cellular activities [5,6,7]. By scanning a designated area of an electrolyte–insulator–semiconductor (EIS) structure with a modulated light beam, spatiotemporal AC photocurrent images with the two-dimensional distribution of analytes are produced [8,9].

To enhance the spatial resolution and photocurrent response, a wide range of semiconductor substrates have been investigated. Silicon on insulator (SOI) [10,11], ultrathin silicon on sapphire (SOS) [12] and semiconductor materials such as amorphous silicon, GaAs [13], GaN [14], TiO_2_ [15] and In-Ga-Zn oxide [16] have been studied. SOS substrates exhibited a high resolution of 1.5 μm with a focused 405 nm laser beam and a resolution of 0.8 μm using a two-photon effect with a 1250 nm femtosecond laser [12]. SOS functionalized with self-assembled monolayers (SAMs) as an insulator has been used for imaging of chemical patterns [17,18,19], microcapsules [20], and yeast cells [21]. Modifying silicon with SAMs terminated with redox active species allowed the imaging of photo-induced redox currents [22].

Recently, ITO-coated glass without any insulator was proposed as a low-cost and robust substrate for photoelectrochemical imaging [23,24]. In the absence of an insulator, the AC photocurrent is largely determined by the anodic oxidation of hydroxide making ITO-LAPS highly sensitive to pH (70 mV/pH). Photocurrent imaging with ITO-LAPS showed a good lateral resolution of 2.3 μm [23] and was confirmed to be sensitive to the surface charge of living cells [24]. ZnO nanorods were used as a substrate for AC photocurrent imaging to monitor the degradation of a thin poly (ester amide) film with the enzyme α-chymotrypsin, also showing great potential in biosensing and bioimaging applications [25]. However, a relatively high applied bias (1.5 V) was required to achieve sufficiently high photocurrents with ITO and ZnO nanorods for two-dimensional imaging, which could possibly interfere with cellular metabolism. Moreover, due to low charge carrier mobility, both ITO and ZnO suffered a dramatic decrease in photocurrent with increasing modulation frequency, resulting in a low working frequency of 10 Hz for imaging. This could consequently limit their application for high-speed imaging, which is required for the investigation of cellular responses.

In this work, InGaN/GaN on sapphire was investigated as a new substrate for AC photoelectrochemical imaging, aiming to solve the above-mentioned problems. InGaN is a semiconductor alloy with a direct band gap that can be tuned from the near-infrared (0.6 eV, InN) to the ultraviolet (3.4 eV, GaN) by adjusting the indium concentration. It has been used widely in developing LEDs [26,27] and photovoltaic devices [28] owing to its strong light emission and absorption and a wide range of band gaps. InGaN has also gained significant attention in photoelectrochemistry. With band edges straddling oxygen and hydrogen redox overpotentials, p-type GaN/InGaN nanowires have been investigated in water splitting [29], having the advantages of high carrier mobility, good chemical stability and band gap tunability. GaN/InGaN nanowires have also been shown to exhibit excellent optochemical and electrochemical sensor performance, achieving the detection of pH [30], oxidizing gases (O_2_, NO_2_ and O_3_) [31] through photoluminescence, and electrochemical detection of nicotinamide adenine dinucleotide (NADH) [32]. In this work, it will be shown that epitaxial layers of InGaN are suitable for photoelectrochemical imaging with good lateral resolution and have great potential in bioimaging applications.

## 2. Experimental Section

### 2.1. Materials

The InGaN/GaN structure was grown on a two-side polished (0001) sapphire substrate in a Thomas Swan 6 × 2” metalorganic vapor-phase epitaxy reactor using trimethyl gallium (TMG), trimethyl indium (TMI), silane (SiH_4_) and ammonia (NH_3_) as precursors, while purified hydrogen and nitrogen were used as the carrier gases. A 40-nm-thick low-temperature (580 °C) GaN nucleation layer was followed by a 100-nm-thick n-type GaN layer deposited at 1060 °C in a hydrogen atmosphere at a constant pressure of 100 Torr. The carrier gas was then switched to nitrogen, the pressure ramped at 300 Torr and the temperature to 770 °C for the growth of the 100-nm-thick n-type InGaN epilayer.

All wet chemicals were purchased from Sigma-Aldrich (Gillingham, UK). All solutions in this work were prepared using ultrapure water (18.2 MΩ cm) from a Milli-Q water purification system (Millipore, Burlington, MA, USA).

### 2.2. Preparation and Characterization of Sensor Chip

The InGaN/GaN structure was cut into 5 mm × 5 mm pieces. These were ultrasonically cleaned with acetone, isopropanol and ultrapure water each for 15 min and blow dried with nitrogen. The InGaN/GaN samples were kept at room temperature before use. The morphology of InGaN/GaN was examined using a scanning electron microscope (SEM, FEI Inspect F, Thermo Fisher Scientific, Hillsboro, OR, USA). Ultraviolet–visible (UV-vis) spectra were obtained using a UV-Vis spectrometer (Lamda 950, PerkinElmer, Seer Green, UK). 

### 2.3. Linear Sweep Voltammetry (LSV)

LSV of InGaN/GaN was carried out in Dulbecco’s Phosphate Buffered Saline (DPBS) solution (pH 7.4) using an Autolab PGSTAT30/FRA2 electrochemical workstation (Windsor Scientific Ltd., Slough, UK). A platinum electrode and an Ag/AgCl (3 M KCl) electrode were the counter electrode and reference electrode, respectively. The scan rate was 10 mV/s. A diode laser (λ = 405 nm, max 50 mW), chopped in 10 s intervals was used as the light source while recording the LSV curves.

### 2.4. Cell Culture

Before seeding cells, InGaN substrates were sterilized with 70% ethanol and rinsed thoroughly with sterilized DPBS solution and blown dry. MG-63 human osteosarcoma cells were cultivated in Dulbecco’s Modified Eagle’s Medium (DMEM, Cat No D6429) supplemented with 10% Fetal Bovine Serum (FBS, Cat No F9665) and 1% penicillin-streptomycin (Cat No P4333) in an air jacketed incubator with 5% CO_2_ at 37 °C with the medium changed every two days. At 70–80% confluence, cells were trypsinized by using Trypsin-EDTA (Cat No T3924), and resuspended in 10% FBS-supplemented DMEM, seeded onto the InGaN surface at a concentration of 2.5 × 10^4^ cells/mL and incubated at 37 °C with 5% CO_2_ for 24 h.

The cell viability was tested using a fluorescence live/dead assay (Thermo Fisher Scientific, Hillsboro, OR, USA, cat. no.: L3224). MG-63 cells were seeded onto two pieces of InGaN (5 mm × 5 mm) assembled in the photoelectrochemical imaging chamber at a concentration of 9.4 × 10^5^ cells/mL and incubated at 37 °C with 5% CO_2_ for 24 h. One InGaN chip was subjected to a raster scan in DPBS while another stayed under ambient conditions for the same time. Then, 0.5 mL of 2 μM calcein AM, 4 μM Ethidium homodimer-1 and 8.12 μM of Hoechst 33342 was used to detect the viability of the cells with and without AC photoelectrochemical imaging. Three different areas in each sample were checked using a fluorescence microscope (Leica DMI4000B Epifluorescence, Leica Microsystems Ltd., Milton Keynes, UK), and cell photos were then processed by Image J software for counting cells.

### 2.5. AC Photocurrent Imaging

**Figure 1:**
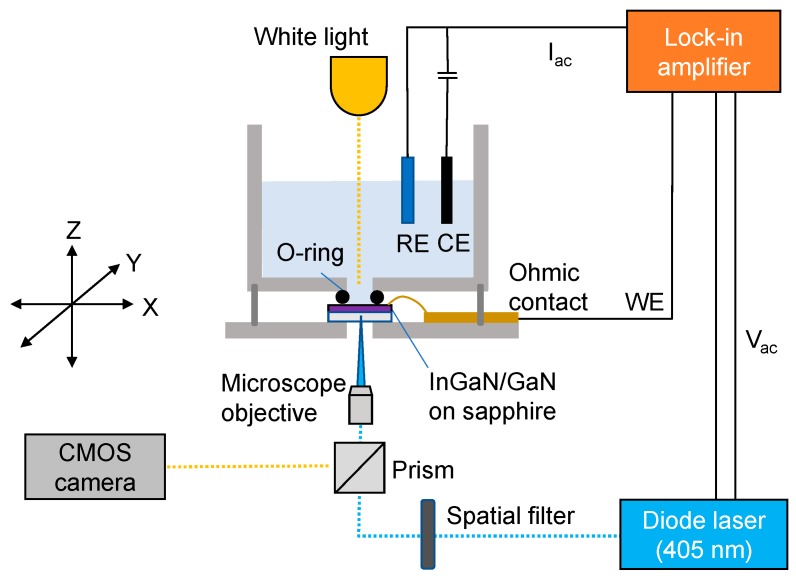
Schematic of the LAPS setup with a 405 nm diode laser to generate photo-induced charge carriers, a lock-in amplifier to measure AC photocurrent, and an X-Y-Z stage to move the electrochemical cell with respect to the laser beam for imaging.

Figure 1 depicts the LAPS set-up used in this work. A diode laser LD1539 (Laser 2000, Huntingdon, UK, λ = 405 nm, max 50 mW) intensity modulated at 1 kHz was used as the light source. The sample chamber was mounted onto an M-VP-25XL XYZ positioning system with a 50 nm motion sensitivity on all axes (Newport, UK). AC photocurrents were measured with an EG&G 7260 lock-in amplifier with a platinum electrode and an Ag/AgCl (3 M KCl) electrode acting as the counter and reference electrodes, respectively. DPBS (pH 7.4) was used as the electrolyte. Optical images of the sensor surface were obtained with a CMOS camera by illuminating the chip surface with white light from the front side. A drop of poly(methyl methacrylate) (PMMA) was deposited on the InGaN surface and dried overnight to obtain a model system for measuring the resolution.

## 3. Results and Discussion

### 3.1. Characterization of InGaN/GaN Epilayers on Sapphire

**Figure 2:**
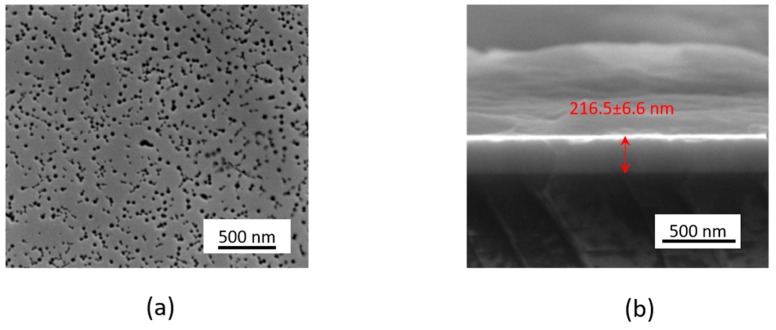
SEM images of InGaN/GaN: (**a**) top view and (**b**) cross-sectional view.

The SEM analysis of the InGaN/GaN structure is presented in Figure 2. The SEM top view in Figure 2a shows the InGaN surface with a high density of pits ((2.26 ± 0.08) × 10^10^ pits/cm^2^) ranging between 20 nm and 50 nm in diameter, as some of the pits have merged. These “V-pits” are well known in InGaN growth and consist of an inverted hexagonal pyramid emanating from a threading dislocation formed at the sapphire/GaN interface. The pits open up during InGaN growth, which takes place at relatively low temperatures [33]. The total thickness of the InGaN/GaN epilayer was about 216.5 ± 6.6 nm, as shown in Figure 2b. Four-probe electrical measurements using soldered indium contacts showed a resistivity of 0.02 Ω·cm due to the n-type conductivity of the epilayers. A photoluminescence (PL) spectral map (Accent RPM2000, exc = 266 nm) of the 2-inch wafer showed a strong emission band centered at 448 ± 2 nm indicating an average indium fraction of ca. 17.5% [34].

**Figure 3:**
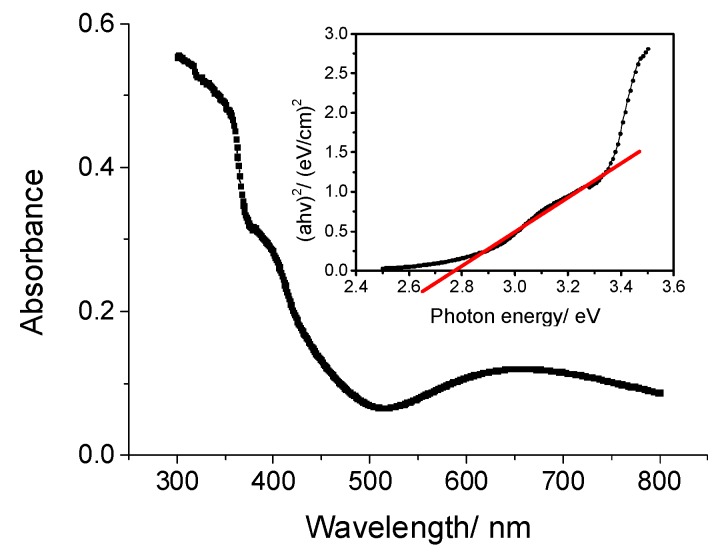
UV-Vis spectrum of InGaN and inset Tauc-plot.

Figure 3 shows the UV-Vis absorption spectrum of InGaN/GaN. From the inset Tauc-plot [35,36], a direct band gap of 2.77 ± 0.03 eV was determined, indicating that the charge carriers in InGaN/GaN are excited at wavelengths ≤ 448 nm, which is in good correspondence with the PL mapping result.

**Figure 4:**
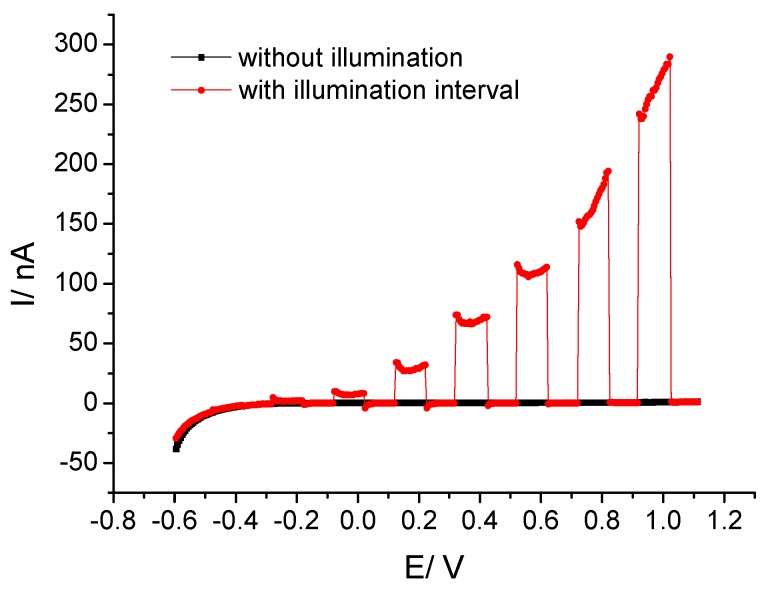
LSV curves of InGaN in the dark and with chopped illumination.

The DC photocurrent response of the InGaN/GaN sample was characterized with LSV. As shown in Figure 4, significant photocurrents were observed at anodic potentials ≥ 0 V. The dark current was negligible compared to the photocurrent. As with ITO substrates, the photocurrent can be ascribed to the oxidation of hydroxide ions in the solution. In contrast to ITO, the InGaN layers show a much lower onset potential of the photocurrent.

**Figure 5:**
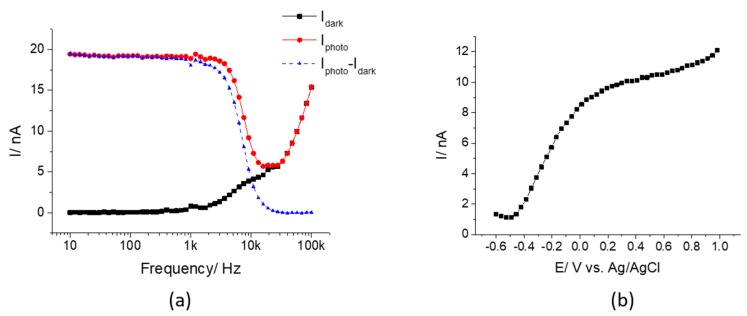
(**a**) Frequency dependence of the AC photocurrent and the background dark current measured at 1.0 V; (**b**) Characteristic *I*−*V* curve of InGaN/GaN measured in pH 7.4 DPBS at 1 kHz with a focused laser beam at 18% maximum intensity.

Figure 5a shows the dependence of the AC photocurrent on the modulation frequency measured at 1.0 V with a focused laser beam. From 10 Hz to 3 kHz, the photocurrent did not change significantly with the frequency, and then it decreased at higher frequencies. Significant photocurrents were obtained up to modulation frequencies of 10 kHz. The photocurrent became negligible at frequencies greater that 20 kHz. In contrast, the AC photocurrent measured with ITO and ZnO previously decreased continuously, with increasing modulation frequency above 10 Hz for ITO [23] and above 30 Hz for ZnO [25], becoming negligible at 7 kHz for ITO and 4 kHz for ZnO. This can be attributed to the significantly higher hole mobilities in InGaN [37] compared to those in ITO [38] and ZnO [39], as low-mobility minority charge carriers will not contribute to the AC photocurrent at high frequencies. In this work, 1 kHz was chosen as the modulation frequency since it could offer high quality images while also demonstrating the potential for high-speed imaging. 

Figure 5b shows the characteristic AC photocurrent−voltage (*I−V*) curve of InGaN/GaN in the voltage range -0.6 V to 1.0 V in pH 7.4 DPBS under the illumination of a focused laser beam (modulation frequency was 1 kHz). It shows that the photocurrent increased with the applied bias, to a value of 12 nA at 1.0 V. Even at 0 V, a photocurrent of 8.5 nA was observed. The low onset potential of InGaN/GaN is in accordance with its low flat band potential [40,41], indicating that the electrode can become depleted by applying a low bias, facilitating the separation of photo-induced charge carriers. Therefore, it provides the possibility for measurements at zero applied bias.

### 3.2. Photoelectrochemical Imaging Using InGaN

**Figure 6:**
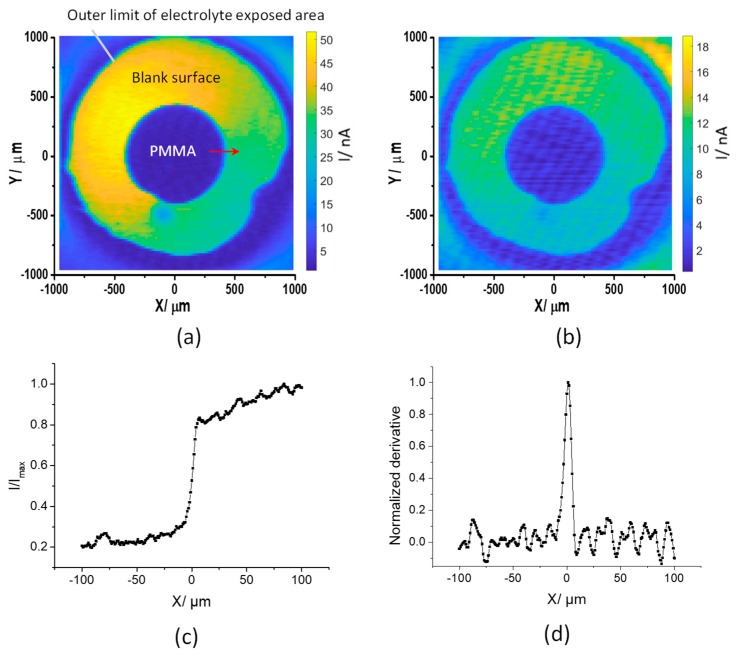
AC photocurrent images of a PMMA dot on InGaN measured at 0.6 V (**a**) and 0 V (**b**); X axis line scan across the polymer edge (indicated by the red arrow in (**a**)) at 0.6 V (**c**) and its corresponding first derivative plot (**d**).

Figure 6a,b shows the photocurrent images of a PMMA dot on the InGaN surface with a modulation frequency of 1 kHz using a focused laser beam at a bias of 0.6 V and 0 V (vs. Ag/AgCl), respectively. The polymer dots were clearly observed in the photocurrent images, with decreased photocurrent values compared to a blank surface area owing to the high impedance of the PMMA dot. The image in Figure 6a shows a significant gradient of the photocurrent across the uncoated area exposed to electrolyte. This can be attributed to the sample not being mounted perfectly perpendicular to the incoming laser beam resulting in a change of the focused laser spot size across the sample. Where applications require imaging over a large area, a tilt module for straightening the sample would have to be integrated into the experimental setup. However, for imaging over small distances, this effect becomes negligible, as will become clear in the next section. The images in Figure 6b and, less obviously, in Figure 6a display a periodic pattern in the photocurrent distribution. It is assumed that this is caused by a striation effect in the InGaN/GaN substrate similar to the one previously observed in silicon [42]. It is worth noting that the ability to image at 0 V will broaden the application of this technique in biological systems, and also possesses an advantage from an energy perspective. To measure the lateral resolution, a photocurrent line scan across the edge of the polymer film was recorded with a focused laser beam and 1 μm step size (Figure 6c). The lateral resolution is derived from the full width at half maximum (FWHM) value of the first derivative of the line [43] (Figure 6d), which is 7 μm for InGaN. This result could be due to a weak adhesion between PMMA and the InGaN surface, thus not giving a steep edge of the polymer, or light scattering within the structure. The diffusion length of minority charge carriers in InGaN should not affect the resolution, as it is less than 200 nm and decreases with increasing indium content [44]. Hence, InGaN is promising for the production of photocurrent images with a higher resolution.

### 3.3. Cell Imaging on InGaN

**Figure 7:**
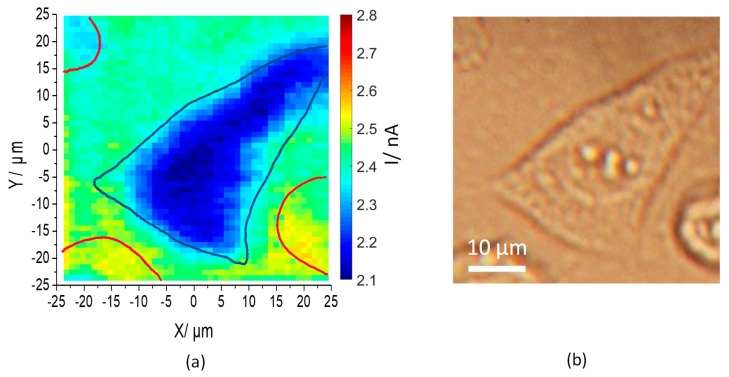
(**a**) AC photocurrent image of a mesenchymal stem cell on InGaN surface (cell shapes from (**b**) superimposed in blue for an attached cell and red for non-attached cells); and (**b**) its corresponding optical image.

Figure 7a shows a photocurrent image of an MG-63 cell seeded on the InGaN surface obtained at a bias of 1.05 V, with a light modulation frequency of 1 kHz. The cell profile is clearly observed, as the photocurrent is smaller in the cell attachment area than on the blank surface. Both the photocurrent image and the corresponding optical image (Figure 7b) show good correlation. Apart from the cell in the center of the image (outline superimposed in blue in Figure 7a), another three cells are visible towards the edges (outlines superimposed in red in Figure 7a). As the latter cells are rounded, it can be assumed that they are not attached to the sensor surface and do therefore not cause a significant change in the local photocurrent. The photocurrent under a cell attached to the semiconductor surface is affected by the narrow gap (> 10 nm) formed between the cell membrane and the surface, as described previously for cells cultured on ITO [24]. The photocurrent is caused by the oxidation of hydroxide. Transport of hydroxide to the surface is hindered by diffusion into the narrow electrolyte gap between cell and surface, thereby reducing the photocurrent under the cell. The negative surface charge of the cell causes an additional reduction in the transport of hydroxide ions to the surface. Hence, a correlation between the photocurrent and the cell surface charge was found [24].

### 3.4. Cell Viability

**Figure 8:**
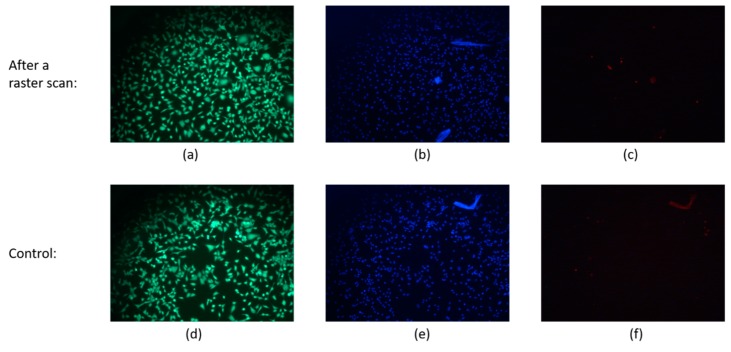
(**a**,**b**,**c**) Fluorescence microscope images of MG-63 cells taken after photocurrent imaging, living cells with intact membranes appeared green, dead cells with collapsed membrane appeared red, and the nuclei of the cells appeared blue. (**d**,**e**,**f**) Images of MG-63 cells that were not subjected to imaging.

To check the invasiveness of InGaN-based AC photocurrent imaging, cell viability for cells with and without AC photocurrent raster scan were tested (Figure 8). Calcein AM can permeate through intact cell membranes and react with the intracellular enzyme esterase, giving an intensely green fluorescence in live cells (excitation/emission 495 nm/515 nm). Ethidium homodimer-1 only passes through disrupted membranes, emitting intense red fluorescence in dead cells upon binding to nucleic acids (excitation/emission 495 nm/635 nm). Hoechst stain is a cell-permeant nuclear counterstain that emits blue fluorescence when bound to dsDNA (excitation/emission 350 nm/461 nm) to determine cell numbers. Results show that 98.92% ± 0.15% MG-63 cells on the surface were viable after a photocurrent raster scan compared to 98.97% ± 0.11% on a control sample, indicating that this imaging technique has no negative effect on the cells.

## 4. Conclusions

An In_0.175_Ga_0.825_N/GaN structure on sapphire was investigated as a substrate for photocurrent imaging without any modification. It showed a considerable photocurrent under illumination with a 405 nm diode laser. Clear photocurrent images of a PMMA dot were obtained with a focused laser beam at 1 kHz modulation frequency, indicating a unique advantage over ITO and ZnO studied previously. In addition, photocurrent imaging at a low bias (0 V) was demonstrated and photocurrent imaging of a cell was achieved, showing a great potential of InGaN for applications in bioimaging and biosensing.
